# Development of a theory-based video-game intervention to increase advance care planning conversations by healthcare providers

**DOI:** 10.1186/s43058-021-00216-8

**Published:** 2021-10-13

**Authors:** Deepika Mohan, Meredith A. MacMartin, Julia S. C. Chelen, Carolyn B. Maezes, Amber E. Barnato

**Affiliations:** 1 Department of Critical Care Medicine, University of Pittsburgh School of Medicine, Pittsburgh, PA USA; 2 Section of Palliative Care, Department of Medicine, Geisel School of Medicine at Dartmouth, Lebanon, NH USA; 3 The Dartmouth Institute for Health Policy and Clinical Practice, Geisel School of Medicine at Dartmouth, Lebanon, NH USA

**Keywords:** Behavioral interventions, Hospitalists, Healthcare providers advance care planning, Attitudes, Motivation, Education

## Abstract

**Background:**

Hospitalization offers an opportunity for healthcare providers to initiate advance care planning (ACP) conversations, yet such conversations occur infrequently. Barriers to these conversations include attitudes, skill, and time. Our objective was to develop a theory-based, provider-level intervention to increase the frequency of ACP conversations in hospitals.

**Methods:**

We followed a systematic process to develop a theory-based, provider-level intervention to increase ACP conversations between providers and their hospitalized patients. Using principles established in Intervention Mapping and the Behavior Change Wheel, we identified a behavioral target, a theory of behavior change, behavior change techniques, and a mode of delivery. We addressed a limitation of these two processes of intervention development by also establishing a framework of design principles to structure the selection of intervention components. We partnered with a game development company to translate the output into a video game.

**Results:**

We identified willingness to engage in ACP conversations as the primary contributor to ACP behavior, and attitudes as a modifiable source of this willingness. We selected self-determination theory, and its emphasis on increasing autonomous motivation, as a relevant theory of behavior change and means of changing attitudes. Second, we mapped the components of autonomous motivation (i.e., autonomy, competence, and relatedness) to relevant behavior change techniques (e.g., identity). Third, we decided to deliver the intervention using a video game and to use the narrative engagement framework, which describes the use of stories to educate, model behavior, and immerse the user, to structure our selection of intervention components. Finally, in collaboration with a game development company, we used this framework to develop an adventure video game (*Hopewell Hospitalist*).

**Conclusions:**

The systematic development of a theory-based intervention facilitates the mechanistic testing of the efficacy of the intervention, including the specification of hypotheses regarding mediators and moderators of outcomes. The intervention will be tested in a randomized clinical trial.

**Supplementary Information:**

The online version contains supplementary material available at 10.1186/s43058-021-00216-8.

Contributions to the literature

- Increasing the frequency of advance care planning conversations (ACP) in the hospital has the potential to achieve national priorities for improving the quality of care provided to patients at the end-of-life.
- Existing interventions have had limited success at changing provider behavior.
- We describe the use of a step-wise, theory-based process of intervention development to increase providers’ autonomous motivation for engaging in ACP conversations.
- This work contributes to the literature by providing a new method of intervention development, and promoting a better understanding of the psychology of health professionals.

## Background

Advance Care Planning (ACP) is an ongoing process of reflection and communication among patients, their family, and the health care team about patients’ preferences, values, and goals of care [1–10]. Participation in these conversations not only stimulates dialogue, but also allows patients to articulate (or potentially to construct) their preferences for care. The products of ACP conversations, such as advance directives, can increase the quality of care for patients at the end-of-life, improve outcomes for family members and caregivers, and decrease costs of care [6, 7].

Unfortunately, across most countries (e.g., USA, Japan, Belgium, the Netherlands), ACP remains rare [1–4]. Hospitalizations offer one opportunity for healthcare professionals to initiate these conversations [5]. However, existing quality improvement efforts, which use text-based education, reminders, incentives, and outreach by opinion leaders, have had variable success at changing behavior in this area [8–10].

Our objective was to develop a theory-based, provider-level intervention to increase the frequency of ACP conversations in hospitals. Behavioral interventions most frequently originate opportunistically—as the product of developer intuition and available resources [11–13]. However, theory-based interventions offer a number of advantages: (1) they have an increased likelihood of success; (2) they allow comparison across domains and populations; (3) they provide an opportunity to test the validity of theory [14–17]. In this paper we describe the process we followed to ensure the mapping of theoretical principles to intervention components during the development process. This process was an amalgam and extension of two well-described methods of building theory-based interventions: Intervention Mapping and the Behavior Change Wheel.

## Methods

### Overview of our process

We identified two dominant strategies in the dissemination and implementation literature for building theory-based interventions: Intervention Mapping and the Behavior Change Wheel [12, 16]. These well-established processes offer clear, systematic guidance for developing a theory-based, rather than theory-inspired, intervention. They start by defining the target behavior, progress to specification of what needs to change, and end with the identification of appropriate behavior change techniques [12, 16–18]. However, they have important limitations. For example, Intervention Mapping lacks a comprehensive taxonomy of behavior change techniques that users can reference when attempting to operationalize behavior change objectives [16]. The Behavior Change Wheel explicitly divorces selection of behavior change techniques from theories of behavior, instead of focusing on the function of the intervention (i.e. to persuade or train) as the driver of technique selection [19]. Most notably, both strategies ignore the complexity of instantiating behavior change techniques into the intervention, and the potential influence that the quality that those processes have on efficacy [12, 16]. For all these reasons, we combined and extended the steps outlined by Intervention Mapping and the Behavior Change Wheel, and summarize our process in Table 1. We describe Phases 1–3 in this manuscript.

**Table 1:** Outline of phases of intervention development and relationship to processes followed in Intervention Mapping and the Behavior Change Wheel

Phase of Intervention Development	Intervention Mapping [13]	Behavior Change Wheel [9]
*Phase 1 – Understand the behavior and specify the target for change*	*Step 1: Logic model of the problem* • Establish and work with a planning group • Conduct a needs assessment • Describe the context for the intervention including the population, setting, and community • State program goals	*Stage 1: Understand the behavior* 1. Define the problem in behavioral terms 2. Select target behavior 3. Specify target behavior 4. Identify what needs to change
	*Step 2: Program outcomes and objectives – logic model of change* • State expected outcomes for behavior and environment • Specify performance objectives for behavioral and environmental outcomes • Select determinants for behavioral and environmental outcomes • Construct matrices of change objectives • Create a logic model of change	
*Phase 2 – Identify theoretical basis for proposed behavior change and select relevant behavior change techniques*	*Step 3: Program design* • Generate program themes, components, scope, and sequence • Choose theory and evidence-based change methods • Select or design practical applications to deliver change methods	*Stage 2: Identify* intervention options 1. Intervention functions 2. Policy categories *Stage 3: Identify content and implementation options* 3. Behavior change techniques
*Phase 3 – Identify mode of delivery, choose design principles to guide selection of intervention components, and develop the intervention.*	*Step 4: Program production* • Prepare plans for program materials • Draft messages, materials, and protocols • Pre-test, refine, and produce materials	*Stage 3: Identify content and implementation options* 4. Mode of delivery
*Phase 4 – Assess the fidelity of intervention delivery*	*Step 5: Program implementation plan* • Identify potential program users • State outcomes and performance objectives for program use • Construct matrices of change objectives for program use • Design implementation interventions	N/A
*Phase 5 – Assess intervention efficacy and test mediators and moderators of the outcome*.	*Step 6: Evaluation plan* • Write effect and process evaluation questions • Develop indicators and measures for assessment • Specify the evaluation design • Complete the evaluation plan	N/A

### Phase 1: Understand the behavior and specify the target for intervention

We reviewed the literature to address two questions: (1) what criteria should providers use when deciding when/whether to have ACP conversations; (2) what barriers and facilitators of the behavior exist [1, 5, 8–10, 20–27]. We found recommendations issued by the Society of Hospital Medicine for providers when making decisions about ACP conversations [5]. To further enrich this information, we performed (a) a Delphi-panel study to generate expert consensus guidelines for ACP conversations in the hospital; (b) semi-structured interviews with a sample of hospital leaders responsible for leading ACP quality improvement efforts across the country. The details of these studies have been published elsewhere [21, 22].

From the literature review, Delphi panel, and interviews, we identified modifiable sources of influence on ACP behavior and selected one key source of influence to target in our intervention. This decision was informed by our assessment of the effectiveness of other interventions, the likelihood of behavior change, the spillover effect of change on other sources of influence, and the ability to measure the influential variable(s).

### Phase 2: Identify theoretical basis of behavior change and select relevant behavior change techniques

Next, we reviewed existing theories of behavior to identify those aligned with the determinants of providers’ behavior [16, 28]. Our decision to tie intervention development explicitly to scientific theories (rather than focusing more generally on intervention function as advocated by the Behavior Change Wheel approach) reflected two considerations: (1) we wanted to exploit the explanatory power of theory, increasing our ability to predict mediators and moderators of the intervention’s efficacy; (2) we wanted to assess the applicability of the theory, thereby allowing us to further our understanding of health professionals’ decision-making. We selected a primary theory based on its alignment with the determinant of behavior selected in Phase 1 and its ability to address our over-arching programmatic goal of ensuring that the intervention would contribute to, rather than undermine, “joy in work” [29].

Once we identified a relevant theory, we reviewed a taxonomy of behavior change techniques—general methods for influencing the determinants of behaviors—to select those most suited to achieve our objective of changing the determinants of providers’ ACP conversations [30].

### Phase 3: Identify mode of delivery, choose framework of engagement to guide selection of intervention components, and develop the intervention

As we progressed to intervention development, we debated the optimal mode of delivery. We considered the following criteria: (1) how to ensure fidelity of intervention delivery; (2) how to maximize the likelihood of effectiveness; (3) how to minimize resources required to disseminate the intervention widely. We screened different modes of delivering behavioral interventions and specifically reviewed the literature on the efficacy of serious games—“games in which education (in its various forms) is the primary goal rather than entertainment”—at improving clinician skill outcomes [31–34]. Once we had selected the mode of delivery (a customized video game), we next considered how to instantiate behavior change techniques into the intervention and how to design game components.

We drew on two bodies of research to identify design principles for our intervention. First, we reviewed the literature on “flow,” defined as a state in which people are so involved in an activity that nothing else seems to matter. Experiences that promote flow traditionally do one of the following: (1) provide competition (e.g., playing sports); (2) give the illusion of controlling chance (e.g., gambling); (3) scramble ordinary perception (e.g., skydiving), or (4) create alternative realities (e.g., reading) [28]. Second, we reviewed the literature on persuasion to identify message features (e.g. content, structure, style) associated with behavior change [35]. These bodies of work overlap in their agreement that narrative, information presented in the form of a story, has the power both to immerse and to persuade and could provide the framework for our behavior change techniques [36, 37].

Next, a multi-disciplinary group of hospitalists, palliative care physicians, and intensivists created game content, identifying five didactic principles that we wanted to communicate during the game (see Additional File 1). Finally, in collaboration with the game development company, with which we had partnered, we selected intervention components (game content, mechanics, and design features) that mapped to domains of engagement. The game development team incorporated intervention components into an existing game, developed previously for a separate project [38].

## Results

**Fig. 1:**
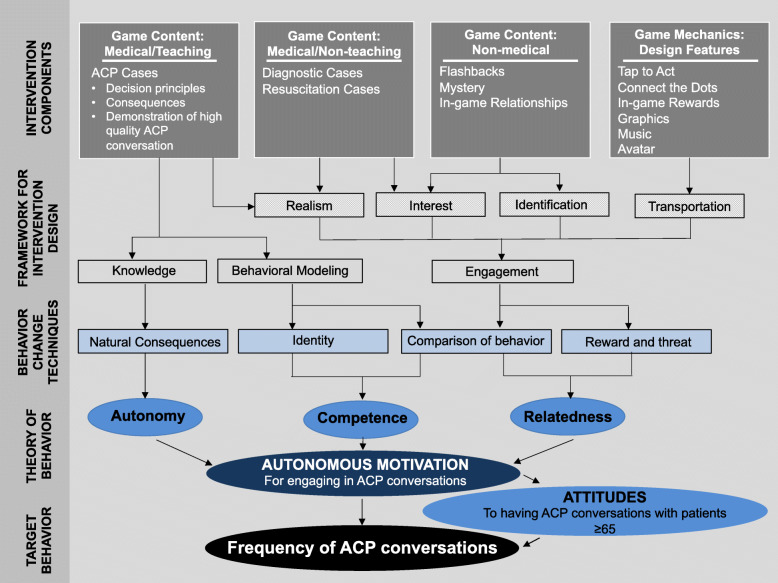
Conceptual model of intervention development. In this figure, we attempt to make transparent how each component of the intervention is intended to intervene on the behavioral process. We denote the relationships among different phases of intervention development with arrows, concrete components of the intervention with rectangles, and theoretical components of the intervention with ovals

We summarize our progression through Phases 1–3 of intervention development in Table 2 and Fig. 1.

**Table 2:** Schematic of process to guide the selection of intervention components

*Phase 1*		*Phase 2*			*Phase 3*
Specify the target behavior	Identify the target determinant of behavior	Identify theory of behavior change	Specify components of theory of behavior change	Select behavior change techniques	Identify design principles
Increase the frequency with which providers have Advance Care Planning (ACP) conversations with hospitalized patients over the age of 65.	Provider attitudes towards ACP conversations	Self-determination theory	Autonomy	Natural consequences • Health consequences • Salience of consequences • Anticipated regret • Emotional consequences	Narrative Engagement Framework: narrative knowledge
			Competency	Comparison of behavior • Demonstration of the behavior • Social comparison Identity • Identification of self as role model • Framing/reframing	Narrative Engagement Framework: behavioral modeling
			Relatedness	Comparison of behavior • Information of other's approval Reward and threat • Social reward	Narrative Engagement Framework: engagement

### Phase 1: Understand the behavior and specify the target for intervention

The Society of Hospital Medicine recommends that hospitalists have ACP conversations for all newly hospitalized patients with “serious illness,” and suggests the use of disease-specific criteria or hospitalists’ response to the surprise question (i.e., “Would you be surprised if this patient died in the next 12 months?”) to screen patients [5]. Based upon our review of clinical practice guidelines, we initially expected that it might be necessary to create and to disseminate rules to help providers recognize patients with “serious illness.” Yet, through a Delphi process, an expert panel recommended that hospitalists have ACP conversations with all patients, over the age of 65 admitted for management of an acute medical problem [21]. These results reframed our behavioral problem from one of recognition (i.e., training providers to detect patients who needed ACP conversations) to one of willingness (i.e., persuading providers to make ACP conversations a priority for all patients over 65 and not just the sickest) [39].

Based on a review of the literature, combined with semi-structured interviews with leaders at acute care hospitals, we identified three categories of barriers that might influence providers’ willingness to have ACP conversations in the hospital: (1) skills—providers felt as if they did not have the communication skills that they needed to have complex, emotionally-laden conversations; (2) attitudes—providers described worry about provoking strong emotions in patients and families with conversations, and the use of avoidance as a strategy to manage their own emotions around challenging topics; and (3) practical barriers—providers identified lack of available time and lack of patient decisional capacity as impediments to ACP conversations [8, 9, 22–27].

Finally, we considered the best target for our intervention. We found no behavioral interventions that effectively modified providers’ attitudes in this domain. We therefore specified that providers’ attitudes towards having ACP conversations with all patients over 65 (and not just those with serious illness) would serve as our intervention target. Our objective was to achieve an increase in the frequency with which providers had ACP conversations (i.e., the target behavior).

### Phase 2: Identify theoretical basis of behavior change and specify relevant behavior change techniques

#### Phase 2a: Identify theory of behavior change

We reviewed multiple theories of behavior that addressed constructs related to attitudes, including beliefs, intention, and motivation: theory of planned behavior, stage theories, social cognitive theory, and theories of self-regulation [16]. Self-determination theory (a theory of self-regulation) appeared to have the greatest alignment with our programmatic goal of ensuring that our intervention promoted, rather than undermined, “joy in work” [40]. In self-determination theory, motivation satisfies three needs: competence (the feeling of mastery of important tasks), autonomy (the feeling of volition associated with action), and relatedness (the feeling of belonging and connectedness with others). These basic psychological needs contribute to health and well-being when met, or pathology and ill-being when unmet. Unlike other theories of behavior (e.g., social cognitive theory), self-determination theory describes a spectrum of motivation, ranging from amotivation (lacking the intention to act) to extrinsic motivation (performing an activity to achieve secondary gain) to intrinsic motivation (performing an activity because it produces inherent satisfaction). As the locus of behavioral control shifts from external to internal, motivation becomes increasingly autonomous or self-determined. Autonomous motivation can occur because the task generates innate satisfaction (intrinsic motivation) or because it aligns with deeply held values (integrated regulation of extrinsic motivation). Interventions that maximize autonomous motivation promote persistence, positive affect, enhanced performance, and greater psychological well-being [41, 42]. Autonomous motivation most closely aligned with the construct of willingness that we sought to target. Moreover, self-determination theory asserts that this type of sustainable motivation “emerges from one’s sense of self and is accompanied by feelings of willingness and engagement.”

#### Phase 2b: Identify behavior change techniques

Michie et al. developed a comprehensive list of 93 distinct behavior change techniques and categorized them into a hierarchical structure using Delphi methods [30]. We reviewed the taxonomy to identify techniques that aligned with our objective of maximizing providers’ autonomous motivation for engaging in ACP conversations. We identified four groups of relevant techniques: (1) providing providers with information about the natural consequences of ACP conversations; (2) allowing providers to make comparisons of the behavior; (3) providing providers with information about reward and threat associated with the behavior; (4) linking the behavior to the providers’ professional identity. We list the techniques we selected in Table 2.

### Phase 3: Identify mode of delivery, choose design principles to guide selection of intervention components, and develop the intervention

#### Phase 3a: Identify mode of delivery

We identified a serious video game as most likely to meet our objectives of a high-fidelity, effective mode of delivery. In addition, although costly to develop, a game would require relatively few resources to disseminate, facilitating the scalability of the initiative [43]. Finally (and pragmatically), we had a game that we could repurpose for this project, which further reduced costs.

#### Phase 3b: Select overarching design principles to guide selection of intervention components

We decided to use the narrative engagement framework to instantiate behavior change principles into the intervention, as it aligned with decisions regarding the behavioral target, the behavior change techniques, and the mode of delivery [44]. Narrative is defined as “talk organized around significant or consequential experiences, with characters undertaking some action, within a context, with implicit or explicit beginning and end points, and significance for the narrator and his/her audience” [45]^.^ Narratives have had great success in health promotion efforts (e.g., reduction of sexually transmitted infections, increase in mammogram screening) as a means of education and persuasion, and may allow the delivery of behavior change techniques in other domains [46, 47]. Narratives achieve their effect through communication of knowledge, behavioral modeling, and engagement [44, 47–49]. We describe key elements of narrative engagement and how they map to our behavioral target in Tables 2 and 3.

**Table 3:** Description of domains of the narrative engagement framework and relationship to selection of intervention components

Domain	Description	Function	Selection of intervention components
Narrative knowledge	Narrative knowledge is defined as information that is presented already integrated within a mental model (defined as a representation of the decision problem)	• Reduces cognitive load required for processing complex information • Reduces counter-arguing provoked by exposure to a new or controversial argument • Increases memorability of the knowledge contained in the story	• Game content – Teaching cases
Behavioral modeling	Behavioral modeling is defined as opportunities to observe others’ behavior	• Allows the development of new rules for one's own actions. • Offers vicarious experience with the consequences of effective and ineffective behavior	• Game content – Teaching cases
Engagement	Engagement is defined as immersion in the health narrative promoted by: • Realism: believable actions and consequences • Interest: intensity of attention attracted to the story • Identification: a feeling of unity with the characters • Transportation: a cognitive or emotional shift in the state of consciousness.	• Increases acceptance of the knowledge embedded in the story • Increases willingness to practice the modeled behaviors • Increases memorability of the experience.	• Game content - Teaching cases (realism) • Game content - Diagnostic cases (interest) • Game content - Resuscitation cases (interest, transportation) • Game content - flashbacks, mystery, in-game relationships (interest, identification, transportation) • Game mechanics (e.g. tap to act) (interest, transportation) • Game design (e.g. music) (transportation) • Game graphics (e.g. color of text bubbles) (interest, transportation)

#### Phase 3c: Develop the intervention

Finally, we worked with a video game company (Schell Games) to develop the game intervention. We used as our template a game that we had previously developed to recalibrate providers heuristics in trauma triage [38]. We kept the art and game mechanics but modified the story and the in-game interactions to deliver the behavior change techniques identified above. We summarize the game components in Table 3 and Fig. 1. We show an example of the game content mapped to behavior change techniques and domains of the narrative engagement framework in Table 4 and in Additional File 1. We include a full description of the final product (*Hopewell Hospitalist*) in Additional File 1.

**Table 4:** We show an example of the game’s medical content, mapped to behavior change techniques (BCT) and the narrative engagement framework (NEF). In this teaching case, Benjamin is a 70-year-old male who presents initially with confusion. He comes to the ED at the insistence of Moira (his sister and primary caregiver). His past medical history is significant for a recent admission for osteomyelitis. Six days after the initial presentation, Benjamin experiences an aspiration event with respiratory decompensation. Based on the dialogue option selected by the player (ACP conversation v. code status elicitation), the case unfolds through an initial encounter, second encounter, and then feedback from an in-game character (Felix – Andy’s boss)

Dialogue option *After examining and speaking with Benjamin and Moira, the player opts for:*	Initial encounter *Demonstration of behavior (BCT)* *Behavioral modeling (NEF)*	Second encounter *Natural consequences (BCT)* *Narrative knowledge and engagement (NEF)*	Feedback from in-game character *Comparison of behavior and reward/threat (BCT)* *Narrative knowledge and engagement (NEF)*
*Advance care planning (ACP)* *conversation*	...Andy: Hmm…how much information about what the future may hold would be helpful hear?” Moira: “Since his wife died, I’m his only family. I would want to know what might be coming.” Andy: “Ok. It looks like he has a pretty serious pneumonia. ... Given that this is his second serious hospitalization and infection in the last little while, I’m worried he might not have the strength to recover.” Moira: “What do you mean?” Andy: “It’s possible he may have more trouble breathing and we’d be faced with a decision about the ICU and life support. If that happened, there is a risk he could die. Moira: “Oh my God. I knew he was weak but just didn’t think he was that sick!” ...Andy: “What are the things that are most important to him?” Moira: “He’s stubborn and never could do anything that wasn’t his idea first. He wants to be out fishing with his son and grandson, and reading those spy novels he loves.” ... Andy: “Could I make a recommendation?” Moira: “Yes, please.” Andy: “Given what you’ve told me about what matters most to Benjamin, I think a short trial of ICU-level care would make sense as long as we think that he could recover sufficiently to return home and live independently.” Moira: “That makes sense to me”	...Andy: “When we talked last time, we decided that Benjamin would be open to a short trial of a breathing machine in the ICU if we get him through the illness and back home. Do you still think that would be what he would want?” Moira: “I’ve been thinking about this ever since we first talked and yes, I do think he would want a chance to get better. But what if he doesn’t?” Andy: “I can hear your worry. If he doesn’t improve in the next few days, then I worry he could die or, if he survives, need a long stay in a rehab facility…” Moira: “He said he’d never go to a nursing home…” Andy: “I remember you saying that. Let’s hope for the best, but if he doesn’t turn around, we will be prepared to shift our focus to comfort measures only.” Moira: “I’m so glad I’ve been telling him how much I love him these last few days.”	Felix: “I hear Benjamin’s sister is having a hard time.” Andy: “Her brother is dying. So yeah. But at least she knows she is doing what he wanted.” Felix: “It’s great that you had the conversation when he came in. Things might have gone differently otherwise.” Andy: “I just figured…70 year old guy, bad pneumonia, second recent admission. Even if he made it through this hospitalization, his risk of needing a nursing home or having a complication was pretty high.” Felix: “I know. It’s so helpful to the family to talk about before a crisis. Waiting until the patient is decompensating or already in the ICU on life support means that no one is prepared. Everyone’s in panic mode. Then families end up racked with guilt later making decisions about withdrawing life support.” Andy: “They don’t tell you about this part in med school – death is a taboo topic! But knowing how good it can feel to really help people through these hard times would have made me more motivated to learn how to have the conversation in med school.”
*Code status elicitation*	Andy: “Do you know if your brother would want CPR?” Moira: “CPR? Like with the paddles? Well yeah, he would want that. I mean otherwise he would just die, right?” Andy: “Right. I don’t think he’ll arrest, but I always like to ask to be sure I know what people would want, you know, in the worst-case scenario. It sounds like he would want us to do everything.” Moira: “Well yeah, do everything you can to help him!”	Andy: “Your brother has a pretty serious pneumonia that we’ve been treating with antibiotics. Unfortunately, it looks like he’s gotten sicker and we may need to move him to the ICU and intubate him. (sharing medical update) Had you ever talked about whether he’d want a breathing machine if he got this sick? Moira: “No, never. This has all happened so fast I just don’t know what to think.” Andy: “Ok, well let’s think together. What are the things that are most important to him?” Moira: “I just don’t know. How can this be happening? You said he would be okay. He was fine when he came in. I just…” Andy: “I know that this is hard, but we need to make some important decisions here.” Moira: “I am never going to have a chance to say goodbye, am I?”	Felix: “I hear the family of the patient Benjamin is having a hard time.” Andy: “Yes. His sister is struggling because they never really spoke about his wishes.” Felix: “But this is his second recent admission, isn’t it?” Andy: “Yes. He was in with osteo a couple of weeks ago.” Felix: “Do you think it would have helped if you started the goals of care conversation earlier?” Andy: “What do you mean?” Felix: “Well, 70 year-old guy who comes in after a recent admission and now has another bad problem? Best case scenario he could have bounced back and made it home, but there was a reasonable risk he’d deteriorate and end up in the unit on the vent. Even if he survives the vent he’s got about a 70% chance of dying in the next year. If you’d had a real sit-down discussion with them about that risk and assessed his goals and preferences, maybe this part wouldn’t be so hard.” Andy: “I guess I just didn’t think it was time yet.” Felix: “When you think ‘it’s time,’ it’s probably already too late. There’s no harm in hoping for the best but preparing for the worst. It can make hard decisions you might have to make later a little easier for both you and the family.”

## Discussion

This paper describes the application of a novel process of intervention development, grounded in principles derived from programs like the Behavior Change Wheel and Intervention Mapping but modified to address pragmatic concerns. Most importantly, we spent significant effort identifying methods to instantiate behavior change techniques into the intervention that would optimize engagement while simultaneously aligning with change objectives. The product of our effort, an adventure video game titled *Hopewell Hospitalist*, is designed to increase providers’ autonomous motivation for ACP conversations and thereby change their attitudes towards, their willingness to engage in, and frequency of having these conversations.

We began intervention development with three goals. Our first goal was to ensure that the intervention had a robust grounding in psychological and behavioral theory. A 2017 scoping review found that only 47% of implementation studies targeting healthcare professionals explicitly used theories of behavior change [50]. Additionally, even when theory served as the foundation of an intervention, the application occurred inconsistently—only 10% of studies with theory-based interventions reported links between behavior change techniques and theoretical constructs [28]. The NIH’s Science of Behavior Change Program recommends that the development of behavioral interventions begin with a clear conceptual model and employ a mechanistic approach to facilitate the testing of mediators and moderators of efficacy [14]. To facilitate this process, we combined and modified the principles from the Behavior Change Wheel and Intervention Mapping, which allowed us to describe transparently our approach to theory-based behavioral intervention development, including the selection of specific intervention components, in a format that will allow other researchers to compare our work to other development approaches.

Our second goal was to maximize engagement. Both the Behavior Change Wheel and Intervention Mapping focus on the identification of relevant theoretical principles with limited consideration of design. However, engagement potentially mediates the efficacy of behavioral interventions [33, 51, 52]. Little is known about design features that definitively produce engagement. The literature on the topic is complicated by differences in definitions of engagement used by researchers in human-computer interaction and behavioral science [51]. Human-computer interaction research focuses on the *experience of engagement*, referencing the subjective feeling of immersion produced by the intervention. In contrast, behavioral science research focuses on the *behavioral consequences of engagement*, such as duration and depth of usage of the intervention. Although correlated, these constructs have important distinctions. For example, an immersive experience can but does not have to increase usage. The majority of efforts to understand engagement with behavioral interventions in healthcare have focused on strategies that promote usability, such as navigation autonomy, message tone, or reminders to use the intervention [52–54]. Less is known about strategies that increase immersion [55, 56]. We plan to test both the success of our strategy to use the narrative engagement framework to induce an immersive experience, and the ways in which the experience and behavioral consequences of engagement mediate the effect of the intervention.

Our third development goal was to ensure that the intervention would foster “joy in work.” Burnout—emotional exhaustion, feelings of cynicism and detachment from work, and a sense of low personal accomplishment—pervades healthcare [57]. Approximately 54% of physicians endorse at least one symptom of burnout, which contributes in turn to increased medical errors, worsened clinical outcomes, and rising costs [29]. Paradoxically, interventions to improve the quality of care can have unintended consequences by having negative effects on professional satisfaction [58]. As a remedy, the Institute of Healthcare Improvement recommends that organizations focus on encouraging staff to appreciate the meaning and purpose of their work [29]. We used this as our guiding principle during the phases of intervention development, attempting to tie ACP conversations to values that attract many providers to the profession (e.g., altruism, relatedness) and therefore promote the integrated regulation of extrinsically motivated behavior.

This project had three main limitations. First, we grounded the intervention in a single theory: self-determination theory [59]. The selection of a different theory (e.g., theory of information processing) or the use of an integrated framework of theories of behavior change (e.g., the Theoretical Domains Framework) might have produced different results. Second, we focused narrowly on provider attitudes and motivation without explicit consideration of other influences on providers’ willingness to engage in these conversations (e.g., time constraints) or ecological moderators of behavior (e.g., hospital norms). Many programs to address these variables already exist, so that our work adds to the portfolio of available interventions. Finally, the intervention that we constructed was multi-dimensional, relying on both behavior change techniques and design elements to affect behavior. These different aspects of intervention design may interact in complex ways that will be difficult to disentangle. Similarly, we anticipate the potential for spillover from our main behavioral target (motivation/attitudes) to other drivers of willingness. For example, the behavioral modeling that we included to foster engagement may influence communication skills. Ideally, intervention design would allow for the systematic manipulation of each component of the intervention to identify the combination that provides the best results [60]. However, this was outside the scope of our budget.

## Conclusions

We developed a novel process for building behavioral interventions to improve the performance of healthcare providers by adapting principles from the dissemination and implementation literature. This process focused on the need to ground the intervention in theoretical principles of behavior, to maximize the experience of engagement, and to foster joy in work. We used it to build a customized video game to increase the frequency of ACP conversations between providers and hospitalized older patients and plan to test the effect of the intervention in a future randomized clinical trial, which will allow us to better understand the success of our theoretical and design principles.

## Supplementary Information

**Additional file 1.**

## Data Availability

*Hopewell Hospitalist* will be made available for public access on the Apple Store once the trial, testing its efficacy, is complete.
