# Supplementary material for: Development of a theory-based video-game intervention to increase advance care planning conversations by healthcare providers
Source: Implement Sci Commun. 2021 Oct 13;2:117. doi: 10.1186/s43058-021-00216-8 (PMC8513300; doi:10.1186/s43058-021-00216-8)
Supplement: Supplementary file 1 — Additional file 1. [file 43058_2021_216_MOESM1_ESM.docx]

**ADDITIONAL FILE 1**

**Description of Hopewell Hospitalist**

*Hopewell Hospitalist* is a tap-and-click adventure video game designed to shift hospitalists’ threshold for inpatient ACP conversations from only occurring when a patient is at high risk for clinical deterioration to occurring for all hospitalized patients over the age of 65, drawing on CMS policy, interviews with stakeholders, and expert consensus. We adapted the art and game mechanics from a previously-tested game, identified key didactic principles based upon a review of the literature and the input of a multidisciplinary team of palliative care physicians, hospitalists, and critical care physicians, and iteratively piloted the game with a series of physician play-testers between June-August 2019. We summarize the didactic principles in **eBox 1**.

In brief, players take on the persona of Andy Jordan, a young hospitalist who moves home after the disappearance of his estranged grandfather, Robert Jordan, and begins a job at a local community hospital. The player has two objectives: to diagnose and treat patients admitted to the hospital, and to solve the mystery of Robert’s disappearance.

Patient cases (medical content) fall into two categories, 'teaching' and 'non-teaching.' Interactions with the ‘teaching' patients are designed to communicate a didactic principle that instantiates the game objective of encouraging players to have ACP conversations with all patients over the age of 65. These patients have a serious illness but are not at the very end-of-life. When players fail to engage in ACP conversations, the patient returns with complications that require additional treatment. Players also receive feedback on their performance from in-game characters (e.g. peers, family members, or their supervisor). The feedback includes factual information about the probability of poor outcomes among patients over 65 who require hospitalization and a reminder about the value of early ACP conversations. In contrast, when players engage in ACP conversations, they subsequently receive an update about the patient’s condition, describing how that ACP improved the care of the patient downstream, and a compliment on their decision-making and communication skills. Teaching patients also provide an opportunity for players to observe best practice principles of a high-quality serious illness conversation modeled on Ariadne Lab’s Serious Illness Conversation Guide. Specifically, when players choose to engage in ACP conversations, the interaction unfolds with Andy asking key questions from the guide and following other best practices (e.g. he pulls up a chair and sits for the conversation). We show the mapping of behavior change techniques and narrative engagement framework domains to these cases in **eTable 1**, and screenshots of an example of feedback in **eFigure 1.**

‘Non-teaching' patients either have a critical, immediately life-threatening illness or a diagnostically challenging problem. These cases were designed to increase challenge levels and associated game-play enjoyment. Players do not receive in-game feedback on their treatment of ‘non-teaching’ patients. Instead, they receive a summary of their performance on all cases at the end of the game that summarizes decisions made on the teaching cases and the accuracy of their diagnoses for the non-teaching cases.

The mystery component of *Hopewell Hospitalist* occurs concurrently with the clinical challenges, and serves to facilitate players’ identification with their character and interest in their task. Players must solve Robert’s disappearance through interactions with other characters, including patients, and their physical environment. Andy Jordan’s background and character are also revealed through these interactions, which are designed to make him and his decisions more appealing and sympathetic.

**eBox 1. Summary of didactic principles**

| *Didactic principles:* All hospitalized patients who are 65 years or older should have an ACP conversation. Each of the 5 teaching cases (see below) embeds supporting didactic principles in the feedback.   - Older adults who require ICU care for mechanical ventilation have ≥70% risk of death or disability at one year, and pre-admission frailty is associated with even higher risk of death or disability after ICU-level care. Assessing goals for treatment can help hospitalists support goal-concordant treatment decision making when/if medical decompensation occurs. - Patients with severe co-morbidities (e.g., cancer, end-stage renal disease) are at high risk of medical decompensation requiring decisions about ICU-level care, yet ≤10% have had documented ACP conversations with their specialists or primary care providers prior to admission. Hospital admission is a fruitful time for ACP conversations and can be an opportunity to discuss hospice eligibility and introduce hospice services. - Patients hospitalized with even a minor clinical problem have a 30% risk of dying within 3 years. Hospitalization can therefore be an opportunity to think generally about values and goals, and therefore attend to ‘life completion’ tasks. - Having an ACP conversation early reduces the emotional distress and decisional conflict experienced by surrogates and patients when/if medical decompensation occurs. - Race should not influence physician decisions to engage in ACP conversations because individual goals and values, not race, affect patient preferences for end-of-life treatment. |
| --- |

**eTable 1. Description of teaching cases, didactic principles, and how we mapped them to behavior change techniques and narrative engagement framework domains.**

| **Patient name and case description** | **Didactic principle** | **Behavior change techniques** | **Narrative engagement framework domains** |
| --- | --- | --- | --- |
| *Benjamin*, a 70 year old man with pneumonia who suffers an aspiration event 6 days after admission with subsequent respiratory decompensation. | Frailty is associated with increased risk of disability after ICU-level care. | - Natural consequences: health consequences, salience of consequences, anticipated regret (code status option), emotional consequences - Comparison of behavior: social comparison, demonstration of behavior (ACP option), information of other's approval - Identity: identification of self as role model (ACP option), framing/reframing (code status option) - Reward and threat: social reward | - Narrative knowledge - Modeling - Engagement |
| *Darrell*, a 71 year old man with multiple underlying comorbidities including ESRD admitted with an NSTEMI. Returns 1 month later with cardiogenic syncope. | Race-based assumptions can be barriers to engaging in ACP. | - Natural consequences: health consequences, salience of consequences, anticipated regret (code status option), emotional consequences - Comparison of behavior: social comparison, demonstration of behavior (ACP option), information of other's approval - Identity: identification of self as role model (ACP option), framing/reframing (code status option) - Reward and threat: social reward | - Narrative knowledge - Modeling - Engagement |
| *Jonathan*, a 75 year old man with stage IV colorectal cancer admitted with GI bleeding from his rectal stump. | Oncologists have low rates of ACP. Responding to emotion can help with ACP. | - Natural consequences: health consequences, salience of consequences, anticipated regret (code status option), emotional consequences - Comparison of behavior: social comparison, demonstration of behavior (ACP option), information of other's approval - Reward and threat: social reward | - Narrative knowledge - Modeling - Engagement |
| *Linda*, a 65 year old woman who is otherwise healthy, admitted with epigastric pain and melena, found to have upper GI bleeding. | Patients can have varying degrees of readiness, but hospitalization can be an opportunity to think about values and goals. | - Natural consequences: health consequences, salience of consequences, anticipated regret (code status option), emotional consequences - Comparison of behavior: social comparison, demonstration of behavior (ACP option), information of other's approval - Identity: identification of self as role model (ACP option) - Reward and threat: social reward | - Narrative knowledge - Modeling - Engagement |
| Helen, an 80 year old woman with a recent hip repair, initially admitted for a PE, who develops significant GI bleeding. | ACP discussions can decrease emotional distress in surrogate decision makers in the long run. | - Natural consequences: health consequences, salience of consequences, anticipated regret (code status option), emotional consequences - Comparison of behavior: social comparison, demonstration of behavior (ACP option), information of other's approval - Identity: framing/reframing (code status option) - Reward and threat: social reward | - Narrative knowledge - Modeling - Engagement |

**eFigure 1.** Screenshots from game in which the main character (Andy) gets feedback from his boss (Felix). The background for the interaction is: Andy treats a 70 year old patient (Benjamin) for pneumonia. The player has the option of having an ACP conversation with the patient or simply checking Benjamin's code status. Six days later Benjamin experiences an aspiration event with respiratory decompensation, at which point Andy has to make treatment decisions with the Benjamin's sister. The quality and content of the second encounter depends on decisions made during the initial visit. Finally, Andy has a meeting with his boss in which they review his performance. The screenshots demonstrate the ways in which we attempted to communicate behavior change techniques such as comparison of behavior (panel 5) and identity (panel 13-15), and narrative engagement principles such as narrative knowledge (panel 7-8). It also shows some of the game mechanics we used to foster engagement including providing dialogue options (panel 12) and the use of color to evoke emotion (panel 13).
